# A time-dependent subdistribution hazard model for major dental treatment events in cancer patients: a nationwide cohort study

**DOI:** 10.1186/s12903-023-02723-7

**Published:** 2023-02-02

**Authors:** Areum Han, Eun-Gee Park, Jeong-Hwa Yoon, Ji-Yeob Choi, Hee-Kyung Park, Seokyung Hahn

**Affiliations:** 1 Interdisciplinary Program of Medical Informatics, Seoul National University College of Medicine, Seoul, Korea; 2 Integrated Major in Innovative Medical Science, Seoul National University Graduate School, Seoul, Korea; 3 Institute of Health Policy and Management, Medical Research Center, Seoul National University, Seoul, Korea; 4 Department of Biomedical Sciences, Seoul National University Graduate School, Seoul, South Korea; 5 Department of Oral Medicine and Oral Diagnosis, School of Dentistry and Dental Research Institute, Seoul National University, 101 Daehak-ro, Jongro-gu, Seoul, 03080 Korea; 6 Department of Human Systems Medicine, Seoul National University College of Medicine, 103 Daehak-ro, Jongno-gu, Seoul, 03080 Korea; 7 Division of Medical Statistics, Medical Research Collaborating Center, Seoul National University Hospital, Seoul, Korea

**Keywords:** Cancer patients, Dental treatment delay, Dental insurance, Health insurance, Cohort analysis, Competing risk analysis, Time-dependent

## Abstract

**Background:**

Dental care in cancer patients tends to be less prioritized. However, limited research has focused on major dental treatment events in cancer patients after the diagnosis. This study aimed to examine dental treatment delays in cancer patients compared to the general population using a national claims database in South Korea.

**Method:**

The Korea National Health Insurance Service-National Sample Cohort version 2.0, collected from 2002 to 2015, was analyzed. Treatment events were considered for stomatitis, tooth loss, dental caries/pulp disease, and gingivitis/periodontal disease. For each considered event, time-dependent hazard ratios and associated 95% confidence intervals were calculated by applying a subdistribution hazard model with time-varying covariates. Mortality was treated as a competing event. Subgroup analyses were conducted by type of cancer.

**Results:**

The time-dependent subdistribution hazard ratios (SHRs) of stomatitis treatment were greater than 1 in cancer patients in all time intervals, 2.04 within 30 days after cancer diagnosis, and gradually decreased to 1.15 after 5 years. The SHR for tooth loss was less than 0.70 within 3 months after cancer diagnosis and increased to 1 after 5 years. The trends in SHRs of treatment events for other dental diseases were similar to those observed for tooth loss. Subgroup analyses by cancer type suggested that probability of all dental treatment event occurrence was higher in head and neck cancer patients, particularly in the early phase after cancer diagnosis.

**Conclusion:**

Apart from treatments that are associated with cancer therapy, dental treatments in cancer patients are generally delayed and cancer patients tend to refrain from dental treatments. Consideration should be given to seeking more active and effective means for oral health promotion in cancer patients.

**Supplementary Information:**

The online version contains supplementary material available at 10.1186/s12903-023-02723-7.

## Background

Dental care for cancer patients is an important issue because diminished masticatory function in cancer patients or survivors can affect their quality of life by increasing stress and depression [1]. While oral complications resulting from cancer or cancer therapies have been relatively under-recognized compared to other complications, the increasing number of cancer survivors has prompted a wider acknowledgement of the need for more active oral management to ensure long-term well-being [2].

The oral complications related to cancer therapy (e.g., chemotherapy, radiotherapy, targeted agents, and immunotherapy) include stomatitis, dental caries, periodontal disease, and dysphagia, with potentially debilitating effects [2, 3]. Stomatitis, which is a common side effect that results from the loss of integrity of the oral mucosa and inflammatory lesions, occurs in 40% of cancer patients who receive chemotherapy, 76% of those who undergo hematopoietic stem cell transplantation, and nearly 100% of those who receive radiotherapy [4, 5].

Pain, the primary morbidity of stomatitis, may lead to significant problems in food intake, causing weight loss and secondary infection due to nutritional deficiencies [6]. Severe stomatitis degrades patients’ quality of life by frequent emergency department visits or hospitalizations and increases the costs of care due to antibiotic administration or prolonged hospitalization [7]. It can also affect the maintenance of cancer treatment by leading to undesirable breaks in radiotherapy or the discontinuation of planned chemotherapy [8, 9]. Patients undergoing radiotherapy targeting the head and neck are particularly susceptible to a significant deterioration in their oral health. The oral morbidities of radiotherapy include increased susceptibility to dental caries, periodontal disease, and oral mucositis during and after treatment, and these may have harmful effects on daily life function and therefore on quality of life [10]. However, previous oral health-related quality of life studies on head and neck cancer patients undergoing radiotherapy have found that patients’ interest in and prioritization of oral health during treatment tended to decrease compared to pre-treatment due to treatment-related side effects [11, 12].

In this nationwide population-based study using the Korean National Health Insurance Service (KNHIS) claims database, we evaluated the occurrence of incident major dental treatment over time after cancer diagnosis in cancer patients compared to a matched sample of the general population comprising individuals who were never diagnosed with cancer during the entire observation period. We also explored how trends in the occurrence of time-dependent events differed by type of cancer.

## Methods

### Data source

We used the Korean National Health Insurance Service-National Sample Cohort (KNHIS-NSC) version 2.0. This database, established in 2016, is based on administrative claims data from one million people (2% of the whole population), representing the 48 million people who held national health insurance or were eligible for medical care in South Korea from 2002 to 2015. The cohort comprises data from all clinics and hospitals, including information on diagnoses and comorbidities, demographic characteristics, prescriptions, medical services (i.e., treatments and procedures), and costs for inpatients (i.e., those admitted to the hospital) and outpatients (i.e., those who received ambulatory care). It also contains insurance eligibility data, including information on participants’ identity and socioeconomic variables such as sex, residential area, type of health insurance, level of income, type and grade of disability registered, birth, and death.

### Ethics statement and guideline

This study followed the Statement of Strengthening the Reporting of Observational Studies in Epidemiology (STROBE) guidelines [13]. The study protocol was approved by the Institutional Review Board (IRB) of Seoul National University College of Medicine/Seoul National University Hospital, Korea (approval no.: E-1911-012-1076). All methods were performed in accordance with the relevant guidelines and regulations approved by the IRB. The requirement for informed consent was exempted by the IRB of Seoul National University College of Medicine/Seoul National University Hospital, Korea since the KNHIS-NSC dataset comprises de-identified secondary data for research purposes in a fully anonymized form. Restricted access to the database for the study purpose was authorized by the National Health Insurance Sharing Service (NHISS) in Korea (Research Management No.: NHIS-2020-2-144).

### Definition of study participants

Cancer patients were defined as individuals for whom at least one claim was made with hospitalization under a main disease code corresponding to cancer according to the 10th revision of the International Statistical Classification of Diseases and Related Health Problems (ICD-10), or three or more claims were made for a main disease code of cancer within a year since 2006. Those who had cancer diagnoses between 2002 and 2005 were excluded. The control group was defined as individuals with no claims for cancer disease codes throughout the entire period. Those who were younger than 6 years at the time of 2006 or had any missing period due to loss of eligibility for the national insurance system during the follow-up period were excluded.

We randomly assigned an individual from the control group to each cancer patient at a 1:1 ratio after matching by age, sex, residence, and income level in the cancer diagnosis year using an exact matching method. In total, 39,625 cancer patients and a matched control group were selected. For each matching set, the cancer patient and the matching participant were followed from the index date when the cancer patient had his or her first cancer diagnosis until when a dental treatment event of interest occurred, the participant died, or the participant was censored.

### Variables under study

Treatment event outcomes were identified from the claims data for four dental diseases: stomatitis, tooth loss, dental caries/pulp disease, and gingivitis/periodontal disease. For the operational definition of outcomes, clinical opinions and definitions validated in previous studies were considered [14–16]. An event of treatment for stomatitis was defined as a case in which at least one claim was made with a relevant disease code. Events for tooth loss, dental caries/pulp disease, and gingivitis/periodontal disease were defined as cases in which at least one claim was made with a relevant disease code together with a disease-related treatment code. The relevant treatment codes for each disease were identified on the basis of the Health Insurance Medical Care report published by the Korean Health Insurance Review and Assessment Service (see Additional file 1: Table S1) [17]. The outcome of death was defined as a case in which beneficiaries lost their qualification due to death identified from the insurance eligibility data. Death was considered as a competing event for the outcomes of major dental treatment events.

### Statistical analysis

For each event of interest, we estimated the relative risk (RR) as a ratio of the proportions of the outcome in the control and cancer patients, and the hazard ratio (HR) was also calculated using the Cox proportional hazard regression method [18].

The proportional hazard assumption was explored by a log-minus-log plot. When the assumption was considered invalid, intervals for varying HRs were determined based on clinical advice and the exploration of the log-minus-log curves for any significant points of intersection.

A time-dependent HR of each treatment event was generated by applying a subdistribution hazard model with time-varying covariates [19], which is an extended Cox model accounting for time-varying covariates as well as time-independent covariates, considering death as a competing risk for the outcomes of treatment events. The subdistribution hazard ratio (SHR) was estimated at each defined interval and 95% confidence intervals (CIs) were computed.

Baseline diseases were considered as covariates for adjustment, and they primarily included diabetes, hypertension, hyperlipidemia, arthritis, osteoporosis, infectious disease, gastrointestinal disease, cardiovascular disease, and cerebrovascular disease. The baseline diseases were defined operationally as cases in which two or more disease-related codes were found within a year prior to the follow-up period. For diabetes, hypertension, and hyperlipidemia, cases where a disease-related code and at least one record of relevant medication prescribed for the disease were found were also included as indicators of baseline disease. A history of any dental disease of interest (i.e., stomatitis, tooth loss, dental caries/pulp disease, and gingivitis/periodontal disease) within a year prior to beginning of the follow-up period were also considered as a baseline disease covariate for adjustment (see Additional file 1: Table S2).

A subgroup analysis was performed by type of cancer in consideration of the cancer treatment site (oral cancer, other head and neck cancer, thyroid cancer, other solid cancer, and blood cancer) (see Additional file 1: Table S3).

All statistical analyses were performed using R version 3.3.3 [20] and SAS Enterprise Guide software (version 7.1; Copyright © 2003 SAS Institute Inc., Cary, NC, USA).

## Results

### Cohort characteristics

There was no significant difference between the groups in age, sex, residence, and income level, which were used as matching variables (*p* = 1.00). No significant differences between groups were found in the history of tooth loss, dental caries/pulp disease, and gingivitis/periodontal disease (*p* = 0.71, *p* = 0.55, and *p* = 1.00, respectively), whereas a significantly higher proportion of cancer patients had experienced stomatitis at baseline (*p* < 0.01). Gastrointestinal disease was present in 39.2% of cancer patients but in 28.5% of the control group (*p* < 0.01). The prevalence of all the other baseline diseases showed differences of less than 3% points between cancer patients and the control group, but the differences were still statistically significant at the 0.05 level (Table 1).

**Table 1:** Cohort characteristics

		N (%)		
		Cancer	Control	*p* value
Sociodemographic variables	Age (mean (SD))	60.03 (14.89)	60.03 (14.89)	1.00
	Sex			1.00
	Male	19,701 (49.72)	19,701 (49.72)	
	Female	19,924 (50.28)	19,924 (50.28)	
	Residence			1.00
	Seoul	7968 (20.11)	7968 (20.11)	
	Metropolitan	9880 (24.93)	9880 (24.93)	
	Province	21,777 (54.96)	21,777 (54.96)	
	Income (quantile)			1.00
	0–3	8854 (22.34)	8854 (22.34)	
	3–6	9970 (25.16)	9970 (25.16)	
	6–9	14,533 (36.68)	14,533 (36.68)	
	9–10	6268 (15.82)	6268 (15.82)	
Baseline systemic and oral diseases	Diabetes	5692 (14.36)	4634 (11.69)	< 0.01
	Hypertension	13,180 (33.26)	12,242 (30.89)	< 0.01
	Hyperlipidemia	4686 (11.83)	4136 (10.44)	< 0.01
	Arthritis	6826 (17.23)	6312 (15.93)	< 0.01
	Osteoporosis	1606 (4.05)	1449 (3.66)	< 0.01
	Infectious	4636 (11.70)	3578 (9.03)	< 0.01
	Gastrointestinal	15,544 (39.23)	11,295 (28.50)	< 0.01
	Cardiovascular	2203 (5.56)	2025 (5.11)	< 0.01
	Cerebrovascular	2230 (5.63)	2081 (5.25)	0.02
	Stomatitis	2434 (6.14)	1886 (4.76)	< 0.01
	Tooth loss	1079 (2.72)	1097 (2.77)	0.71
	Dental caries	5368 (13.55)	5310 (13.40)	0.55
	Gingivitis	1681 (4.24)	1682 (4.24)	1.00

## Major dental treatment events and mortality and time-dependent hazard ratios

The proportion of stomatitis treatment events was significantly higher in cancer patients than in the control group (RR = 1.15; 95% CI 1.11–1.18), whereas the proportions of treatment events for tooth loss, dental caries/pulp disease, and gingivitis/periodontal disease were significantly lower in cancer patients (RR = 0.74; 95% CI 0.70–0.77, RR = 0.85; 95% CI 0.84–0.87 and RR = 0.81; 95% CI 0.80–0.82, respectively). The incidence of death in the cancer patients was 4.5 times higher than that in the control group (RR = 4.58; 95% CI 4.41–4.76). As analyzed in terms of time-to-event, the probability of treatment event occurrence for dental caries/pulp disease and gingivitis/periodontal disease in cancer patients became similar but slightly higher than those in the control group, and the estimated hazard ratios were statistically significant (HR 1.10; 95% CI 1.07–1.13 and HR 1.03; 95% CI 1.01–1.05, respectively). The hazard ratios for stomatitis, tooth loss, and death appeared in consistent directions to what the risk ratios showed (HR 1.52; 95% CI 1.46–1.57, HR = 0.95; 95% CI 0.91-1.00 and HR 5.59; 95% CI 5.36–5.83, respectively) (Table 2).

**Table 2:** Relative risks (RRs) and hazard ratios (HRs) for cancer patients compared to the control group

Type of event	N (%)		RR (95% CI)*	HR (95% CI)*
	Cancer	Control		
Stomatitis	7208 (18.19)	6296 (15.89)	1.15 (1.11–1.18)	1.52 (1.46–1.57)
Tooth loss	3006 (7.59)	4087 (10.31)	0.74 (0.70–0.77)	0.95 (0.91–1.00)
Dental caries/pulp disease	11,867 (29.95)	13,892 (35.06)	0.85 (0.84–0.87)	1.10 (1.07–1.13)
Gingivitis/periodontal disease	16,426 (41.45)	20,292 (51.21)	0.81 (0.80–0.82)	1.03 (1.01–1.05)
Death	12,514 (31.58)	2732 (6.89)	4.58 (4.41–4.76)	5.59 (5.36–5.83)

*All results are statistically significant at the 0.01 level

**Fig. 1:**
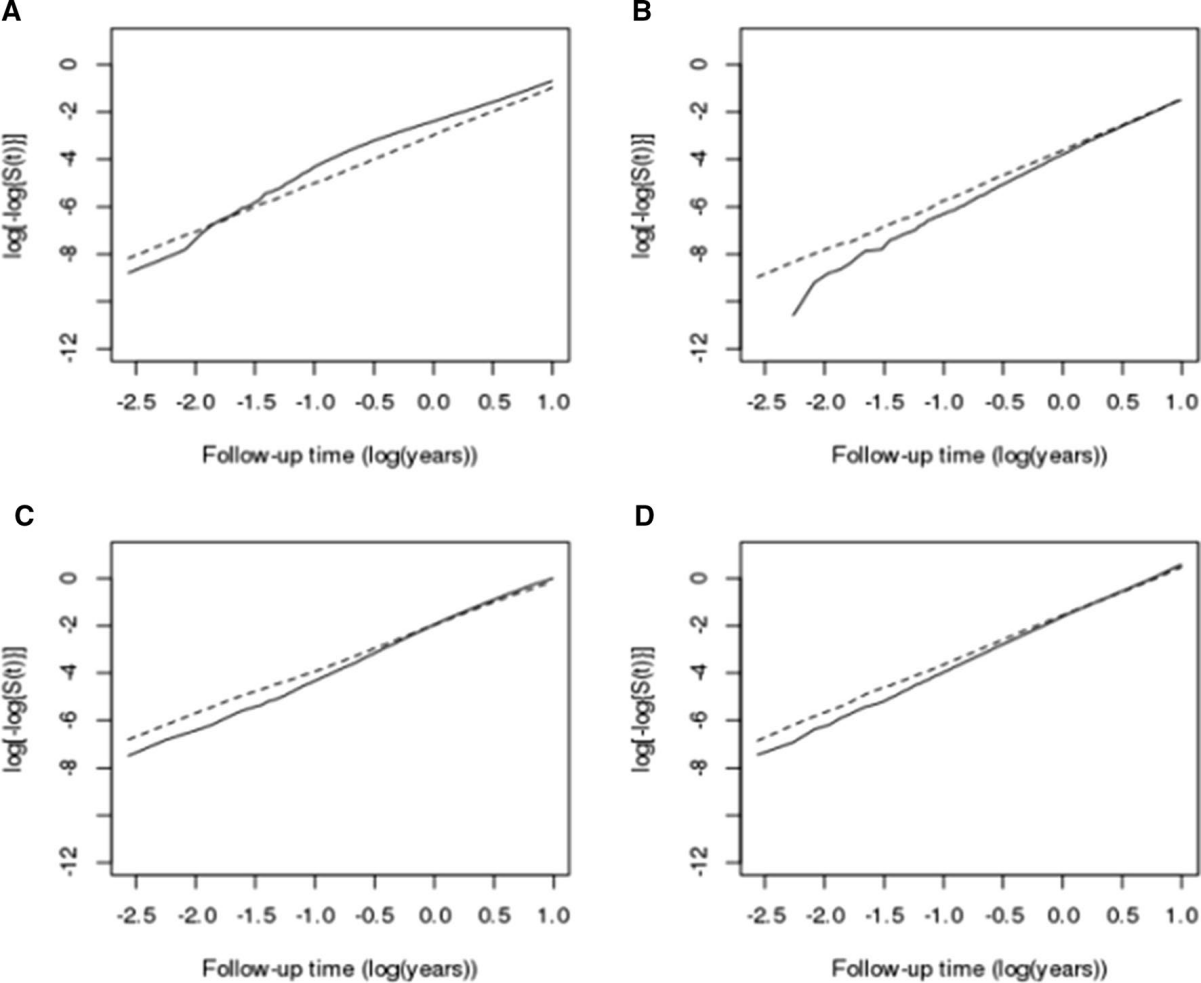
Log-minus-log plots for time to major dental treatment events. Each plot represents stomatitis (**A**), tooth loss (**B**), dental caries/pulp disease (**C**), and gingivitis/periodontal disease (**D**). The solid line shows cancer patients, and the dotted line corresponds to the control group. Follow-up time is expressed as log(years)

In log-minus-log plots, the graphs of cancer patients and the control group intersected for every dental disease of concern, which indicated that the assumption of a constant HR over time did not hold (Fig. 1). Mortality as a competing event for each dental disease was at least twice as high in cancer patients compared to that in the control group in all time intervals, and the highest excess risk was observed in the period from 30 days to 3 months, with a 16-fold difference (Table 3).

**Table 3:** Time-dependent subdistribution hazard ratios (SHRs) of major dental treatment events considering death as a competing event

Outcome	Time	SHR (95% CI)	
		Event of interest	Death before event
Stomatitis	< 30 days	2.04 (1.60–2.58)	6.26 (5.07–7.73)
	30 days to 3 months	1.95 (1.41–2.69)	13.60 (11.04–16.75)
	3 months to 1 year	1.47 (1.10–1.96)	9.43 (7.86–11.31)
	1 year to 3 years	1.23 (1.17–1.30)	5.28 (4.96–5.62)
	3 years to 5 years	1.12 (1.05–1.19)	2.59 (2.38–2.83)
	> 5 years	1.15 (1.04–1.28)	1.98 (1.68–2.32)
Tooth loss	< 30 days	0.65 (0.50–0.84)	7.36 (6.64–8.16)
	30 days to 3 months	0.54 (0.43–0.68)	15.83 (14.79–16.94)
	3 months to 1 year	0.76 (0.69–0.84)	10.27 (9.76–10.81)
	1 year to 3 years	0.91 (0.85–0.98)	5.54 (5.26–5.84)
	3 years to 5 years	0.91 (0.85–0.99)	2.69 (2.50–2.89)
	> 5 years	0.97 (0.89–1.07)^ns^	2.02 (1.84–2.21)
Dental caries/pulp disease	< 30 days	0.83 (0.74–0.92)	5.83 (5.23–6.48)
	30 days to 3 months	0.78 (0.70–0.86)	12.61 (11.72–13.57)
	3 months to 1 years	1.02 (0.98–1.06)^ns^	8.60 (8.17–9.05)
	1 years to 3 years	1.09 (1.05–1.13)	5.16 (4.89–5.45)
	3 years to 5 years	0.97 (0.93–1.02)^ns^	2.68 (2.47–2.90)
	> 5 years	0.93 (0.87–0.99)	2.24 (2.01–2.49)
Gingivitis/periodontal disease	< 30 days	0.80 (0.74–0.87)	5.52 (5.00–6.10)
	30 days to 3 months	0.75 (0.71–0.80)	12.00 (11.27–12.78)
	3 months to 1 years	0.90 (0.87–0.93)	8.48 (8.05–8.93)
	1 years to 3 years	0.95 (0.92–0.98)	5.50 (5.20–5.81)
	3 years to 5 years	0.95 (0.91–0.99)	2.90 (2.67–3.15)
	> 5 years	1.09 (1.04–1.15)	2.53 (2.27–2.82)

All results are statistically significant at the 0.05 level unless indicated as ‘ns’

**Fig. 2:**
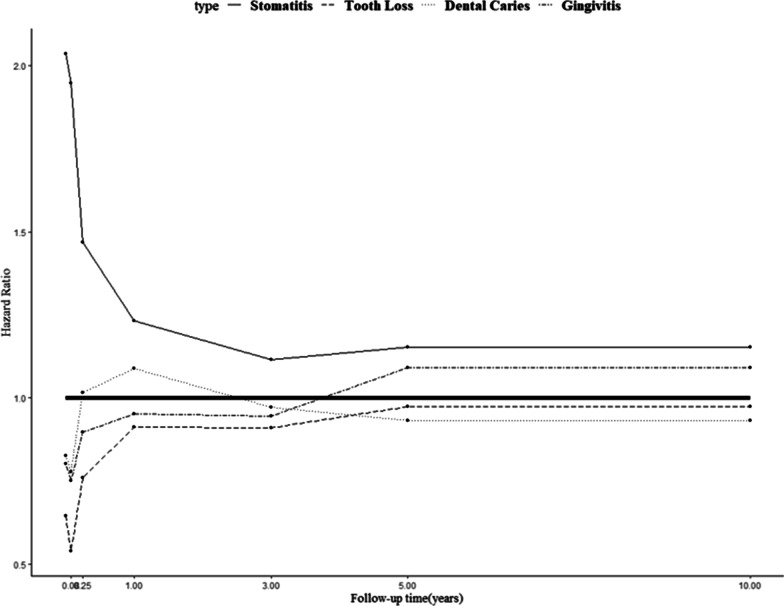
Time-dependent subdistribution hazard ratios (SHRs) of major dental treatment events. The time-dependent SHRs are expressed by points according to the follow-up period. The mortality was considered as a competing event. The bold line represents a hazard ratio of 1.0, indicating the same risk between cancer patients and the control group

The results of the SHR of a treatment event for each dental disease, considering death as a competing event, are presented in Table 3; Fig. 2. The probability of stomatitis treatment event occurrence was greater in cancer patients than in the control group in all time intervals (SHR > 1), with statistical significance at 0.05 level. The SHR was 2.04 (95% CI = 1.60–2.58) within 30 days after cancer diagnosis and gradually decreased to 1.15 (95% CI 1.04–1.28) after 5 years.

The probability of treatment event occurrence for tooth loss was lower in cancer patients than in the control group in all time intervals (SHR < 1), and the SHR was less than 0.7 within 3 months after cancer diagnosis (0.65; 95% CI 0.50–0.84 and 0.54; 95% CI 0.43–0.68 before and after 30 days, respectively) and increased to 1 after 5 years. The estimated SHRs until 5 years after cancer diagnosis were statistically significant at the 0.05 level.

The probability of treatment event occurrence for dental caries/pulp disease was lower in cancer patients. The SHRs were 0.83 and 0.78 within 30 days and 3 months after cancer diagnosis, respectively, with statistical significance at the 0.05 level (95% CI 0.74–0.92 and 0.70–0.86, respectively). The SHR then increased to 1.09 for 1–3 years and was statistically significant at the 0.05 level (95% CI 1.05–1.13).

The SHRs of a treatment event for gingivitis/periodontal disease were 0.80 and 0.75 within 30 days and 3 months after cancer diagnosis, respectively (95% CI 0.74–0.87 and 0.71–0.80, respectively). They gradually increased thereafter, reaching 1.09 after 5 years (95% CI 1.04–1.15). The SHRs were statistically significant at the 0.05 level for all time intervals.

### Subgroup analysis by cancer type

The results for the time-dependent SHR of the treatment event for each dental disease by cancer type are presented in Supplementary Figures and Tables (see Additional file 1: Figs. S1–S4 and Additional file 1: Table S4). The probability of stomatitis treatment event occurrence in cancer patients was higher than in the control group for oral and other head and neck cancers, as well as for blood cancer, in the first 3 months after cancer diagnosis, with SHRs exceeding 3.76. The SHRs were 1.71–2.14 in the same period for thyroid and other solid cancers. The SHRs rapidly decreased until 1 year after cancer diagnosis for all cancer types apart from blood cancer, which showed a relatively slight decrease until 3 years.

Unlike the overall trend in treatment events for tooth loss over time, the probability of event occurrence was greater in patients with oral and other head and neck cancers in the first 30-day period after cancer diagnosis, and the SHRs were 1.94 and 5.12, respectively (95% CI 0.65–5.75 and 2.30-11.42, respectively).

The time-dependent SHRs of treatment events for dental caries/pulp disease by cancer type suggested that the magnitude of reduction in treatment events in cancer patients was larger for blood cancer within 3 months after cancer diagnosis. The SHRs were 0.48 and 0.52, with statistical significance at the 0.05 level, before and after 30 days, respectively (95% CI 0.26–0.88 and 0.34–0.79, respectively). However, unlike the overall trend, the probability of event occurrence was significantly higher in patients with other head and neck cancer in the first 30-day period after cancer diagnosis (SHR = 1.71; 95% CI 1.02–2.87).

Similarly to the results for dental caries/pulp disease, the reduction of treatment events for gingivitis/periodontal disease showed a greater magnitude in patients with blood cancer, particularly in the early phase (within 3 months after cancer diagnosis), with SHRs of 0.48 and 0.40 before and after 30 days, respectively (95% CI 0.29–0.78 and 0.27–0.59, respectively). A significantly greater probability of treatment event occurrence was also noted in patients for other head and neck cancer in the first 30-day period after cancer diagnosis (SHR 2.16; 95% CI 1.49–3.11). In addition, the probability was greater in thyroid cancer patients in all time intervals.

## Discussion

In this study, for the first time, we compared the proportion of major dental treatment events in cancer patients after the diagnosis of any type of cancer with that in the general population using claims data. The proportion of individuals who received stomatitis treatment was significantly higher in cancer patients, while cancer patients showed significantly lower proportions of treatment event for tooth loss, dental caries/pulp disease, and gingivitis/periodontal disease than the general population. In consideration of the much higher mortality rate in cancer patients and the inappropriateness for censoring the deaths that occurred prior to dental treatment, we conducted a competing risk analysis to calculate adjusted HRs. Since the HRs were not constant over time, we estimated time-dependent SHRs. Stomatitis treatment events were observed to have occurred with a greater probability in cancer patients than in the control group for the entire observation period, while those of other dental treatment events were lower in cancer patients until 3 months after cancer diagnosis and then increased, eventually approaching 1. Some differences in the trends were also observed in the subgroup analysis by types of cancer.

The greater occurrence of treatment events for stomatitis in cancer patients in all time intervals, particularly within 3 months after cancer diagnosis, is probably explained by the fact that stomatitis is managed as a major oral complication of cancer therapy. Pain due to stomatitis was found to be the most prevalent (17%) need requiring dental treatment among cancer patients [21], and a preoperative oral examination is performed when preparing a treatment plan for patients undergoing chemotherapy following surgery to help minimize pain [22]. However, the operative definition used to detect cases of stomatitis in this study was not restricted to cancer-related stomatitis.

Apart from stomatitis, the probability of treatment event occurrence in other major dental diseases was significantly lower in cancer patients within 3 months after cancer diagnosis, and the trend of a lower probability of treatment event occurrence was observed for up to 1 year for tooth loss and gingivitis/periodontal disease. Cancer patients generally experience physical damage due to surgery in the course of cancer treatment, limitations in daily life and social activities due to secondary damage caused by chemotherapy or radiotherapy, and deterioration of physical function [1, 23]. Due to these activity restrictions, the treatment of other chronic diseases is often delayed, which may encompass delays in visits to dental clinics and the treatment of gingivitis and periodontal diseases.

The SHR of treatment events for gingivitis/periodontal disease increased and reached a value greater than 1 after 5 years. A reason for this may be that treatment for periodontal disease, which was neglected during the cancer treatment, was actively attempted after the 5-year period that implies cancer survivorship. Alternatively, this trend may be due to the increased incidence of periodontal disease caused by the progression of dental caries during cancer therapy because of delays in dental treatment.

Oral complications of cancer therapy are associated with oncological treatment modalities, which commonly include surgical resection, chemotherapy, radiotherapy, and hematopoietic stem cell transplantation (HSCT) [2, 10]. These therapies are administered alone or in combination and either cause direct harm to the tissue of oral structures or indirectly damage them through their toxicity. Since the usage of treatment modalities is multifaceted, we explored a subgroup analysis by types of cancer. We first separated blood cancer from the others due to the specific treatment modalities, such as HSCT, used for blood cancer. Head and neck cancers were separately explored from other solid cancers because their anatomical location is close to the oral cavity. We also tried to subdivide head and neck cancers into oral cancer, thyroid cancer, and others since we were initially interested in examining whether the state of dental care in oral cancer patients, who could have direct damage to dental structures during cancer treatment, is different from that of patients with other head and neck cancer, and the categorization of thyroid cancer as a head and neck cancer is disputable [24, 25]. However, differences in tendencies by subtypes of head and neck cancers were not clearly observable, which may have been mainly due to the insufficient number of cases in these subcategories.

A subgroup analysis by cancer type suggested that, unlike the overall trend, the probability of event occurrence for tooth loss was significantly higher in head and neck cancer patients in the early stage after cancer diagnosis. In the course of treating head and neck cancer, extraction tends to be recommended if teeth interfere with the surgical site or the prognosis after radiotherapy is expected to be poor [26], which may be an explanation for this finding. The high incidence of oral complications in patients with head and neck cancer results from the location of the main treatment site. While the probability of stomatitis treatment event occurrence was higher in cancer patients in the early stage after cancer diagnosis for all types of cancer, the probability was particularly higher in patients with head and neck cancer. Stomatitis caused by radiotherapy occurs in 60% of patients with head and neck cancer receiving standard radiotherapy [2].

The number of study subjects varied substantially across different cancer types, resulting in a difference in the precision of the estimations of outcomes by cancer type (see Additional file 1: Table 3). The numbers were less than 1000 for oral cancer and other head and neck cancer, and the confidence intervals obtained in those groups were relatively wide. This was the reason for the non-significance of the SHR of 1.9 for tooth loss treatment in oral cancer patients in the early stage after cancer diagnosis. Nevertheless, the high SHR of 5.1 found in other head and neck cancer patients was still highly significant.

The KNHIS-NSC database was established as a population-based cohort by the National Health Insurance Service (NHIS) of Korea—that is, Korea’s universal coverage health insurance system for all citizens—for the purpose of providing public health researchers and policy makers with a nationally representative sample on all beneficiaries of the NHIS, who comprise more than 97% of the entire Korean population. The one million sample cohort was selected from the target population by systematic stratified random sampling with proportional allocation within strata that were defined based on age group, sex, participants’ eligibility status, and income level. The participants were followed up until the endpoint of the cohort unless their eligibility was disqualified due to death or emigration. The cohort was then refreshed annually during the cohort years by adding a representative sample of newborns across all strata to compensate for the number of subjects who dropped out due to disqualification. The sample’s representativeness was evaluated by examining the sample’s average total annual medical expenses compared to the population average in every stratum and by comparing the cohort with the population according to residence distribution across 16 regions in Korea [27].

Since this study used a national health insurance claims database based on the disease and treatment codes generated after visiting medical institutions, the identified times do not accurately reflect when the disease actually occurred. For example, clinic visits for chronic diseases such as gingivitis/periodontal disease do not necessarily indicate disease occurrence. Besides, cases would be missing if patients did not visit a medical institution for treatment. Such issues are well recognized problems in research using claims data. However, it was not our main intention to evaluate the risk of the disease itself; instead, our outcome of interest was defined as an occurrence of a “treatment event” with a focus on comparing the frequency of major dental treatments received between cancer patients and the general population. The outcome was defined as the occurrence of a treatment event rather than disease to facilitate its interpretation as a consequence that reflects participants’ interest in and prioritization of the treatment of dental disease. Concerning treatment events for tooth loss, a particular event did not refer to an event for treating tooth loss, but instead denoted instances when an extraction treatment was administered when it was needed (i.e., a claim for extraction was made with a disease code indicating a state of tooth loss due to disorders of teeth and supporting structures).

There may be some issues in defining stomatitis, that could be identified by using various categories. We defined stomatitis based on disease codes that included not only recurrent aphthous stomatitis, but also stomatitis by other underlying pathologies such as neutropenic ulcerations, herpetic infections, and candidiasis in the oral area. The national claims data do not expose sensitive diseases such as sexually transmitted diseases to prevent the possibility of identification. Therefore, the codes for herpetic infections and candidiasis would not capture herpes type 2 or candida vaginitis in the data. Nevertheless, the diagnosis may still be considered to have been broadly defined. Therefore, treatment events for stomatitis might have been measured at a higher frequency than the actual incidence in both groups.

The discrepancy between real practice and the records in claims databases is a well-known drawback of the analysis of claims data. The process of constructing data for our analysis, including the selection of subjects, also relied on an examination of disease and treatment codes in the database. Clinicians occasionally input inaccurate codes when filling out insurance claims. For example, in dentistry, it is customary to use “chronic periodontitis” when claiming benefits [14], which may result in overestimation of periodontal disease incidence. However, since this issue would apply equally to cancer patients and the control group, ratios (as a relative measure) are less susceptible to potential bias.

Cancer mortality has decreased by more than 30% over the past 30 years [28], and accordingly, the need for dental treatment for cancer survivors is increasing [29]. In Korea, for instance, 34.8% and 36.6% of cancer survivors suffered from mastication problems and periodontal disease, respectively, and 15.9% needed dentures [30]. However, clinical guidelines for dental care for cancer patients are still insufficient and policy decision-making regarding insurance coverage is needed [30–33].

Cancer survivors generally experience financial difficulties, and those who reported financial problems were more likely to experience delays in dental and medical treatment [34, 35]. Even in countries where primary and secondary medical care services are supported through a national health insurance system, although the financial burden of cancer treatment is largely covered by the national health insurance system, the allocation of finances for dental care remains neglected [36, 37]. For example, in Korea, cancer patients are subject to a special coverage system according to which they pay only 5% of the total costs of medical care for 5 years after the date of registration as a cancer patient [38]. However, national health insurance coverage applied to only 31.9% of dental treatments in 2015 [39], even though the coverage for certain dental treatments for the elderly has gradually been expanded, including for dental implants [40]. Our study results suggest that cancer patients tend to refrain from dental treatments, implying that consideration should be given to seeking more active and effective means for oral health promotion in cancer patients. One of the potential suggestions could be extending the national health insurance plan to include dental treatment for oral complications from cancer therapy and covering dental care as a part of healthcare management for cancer survivors. Additionally, well-established programs for oral and dental care in patients receiving cancer therapy should be provided to facilitate full information-sharing regarding the need for dental consultations with healthcare professionals and patients. Furthermore, an effective collaboration system between medical doctors and dentists throughout the course of cancer therapy should also be promoted.

## Conclusion

Apart from treatments that are associated with cancer therapy, dental treatments in cancer patients are generally delayed. There are several potential reasons for the underdevelopment of dental treatment in cancer patients, in relation to issues in prioritization for cancer therapy over dental treatments as well as the healthcare system. Consideration should be given to seeking more active and effective means for oral health promotion in cancer patients.

## Supplementary Information

**Additional file 1:** Supplementary tables and figures.

## Data Availability

Release of the data by the researchers is not permitted. The datasets supporting the findings of this article can only be accessed through the NHISS’ designated network system by any authorized researcher after a formal application to the official website of NHIS data sharing service (https://nhiss.nhis.or.kr/bd/ab/bdaba000eng.do).

## Abbreviations

RR: Relative risk
HR: Hazard ratio
SHR: Subdistribution hazard ratio
CI: Confidence interval
ICD-10: 10th revision of the International Statistical Classification of Diseases and Related Health Problems
KNHIS: Korean National Health Insurance Service
NHIS: National Health Insurance Service
IRB: Institutional Review Board
NHISS: National Health Insurance Sharing Service
KNHIS-NSC: Korean National Health Insurance Service-National Sample Cohort
HSCT: Hematopoietic stem cell transplantation
