# Supplementary material for: A time-dependent subdistribution hazard model for major dental treatment events in cancer patients: a nationwide cohort study
Source: BMC Oral Health. 2023 Feb 2;23:64. doi: 10.1186/s12903-023-02723-7 (PMC9896767; doi:10.1186/s12903-023-02723-7)
Supplement: Supplementary file 1 — Additional file 1: Supplementary tables and figures. [file 12903_2023_2723_MOESM1_ESM.docx]

**Supplementary file**

Supplementary Table 1. Disease codes for dental diseases

| Dental disease | ICD-10 | Treatment code |
| --- | --- | --- |
| Stomatitis | K12, A69.0, B00, B37 | - |
| Tooth loss | K08 | U4412, U4413, U4414 |
| Dental caries/pulp disease | K02, K04 | U0001, U0002, U0010, U0020, U0050, U0060, U0074, U0101, U0116, U0121, U0126, U0210,  U0131-U0138, U0151-U0154, U0200 |
| Gingivitis/periodontal disease | K05 | U1010, U1020, U1030, U1040, U1051, U1052, U1060, U1071, U1072, U1081, U1082, U1100, U1131, U1132, U2231, U2232, U2233, U2240,  U4412, U4413, U4414 |

Supplementary Table 2. Disease codes for baseline diseases

| Baseline disease | ICD-10 |
| --- | --- |
| Diabetes | E11-E14 |
| Hypertension | I10-I13, I15 |
| Hyperlipidemia | E78 |
| Arthritis | M15-M19, M47, M05-M06 |
| Osteoporosis | M80-M81 |
| Infectious disease | A00-B99 |
| Gastrointestinal disease | K25-K31, K50-K52 |
| Cardiovascular disease | I20-I25, I50 |
| Cerebrovascular disease | G45-G46, I60-I69 |

Supplementary Table 3. Disease codes for cancer type and the number of participants

| Cancer type | ICD-10 | N |
| --- | --- | --- |
| Oral | C00-C06, C430-C440, C462 | 886 |
| Other head and neck | C07-C14, C30-C32, C410-C411, C432-C434, C442-C444, C470, C490, C760 | 911 |
| Thyroid | C73 | 6362 |
| Blood | C81-C96 | 1507 |
| Other solid | Other C code | 29959 |
| Total | C00-C97 | 39625 |

Supplementary Figure 1. Time-dependent SHRs of treatment events for stomatitis considering death as a competing event depending on cancer type.

**Supplementary 4. Time-dependent hazard ratios of treatment events for stomatitis considering death as a competing event depending on cancer type as expressed by points according to the follow-up period.** Cancer types are illustrated by different-colored lines. The bold line represents a hazard ratio of 1.0, indicating the same risk between cancer patients and the control group.


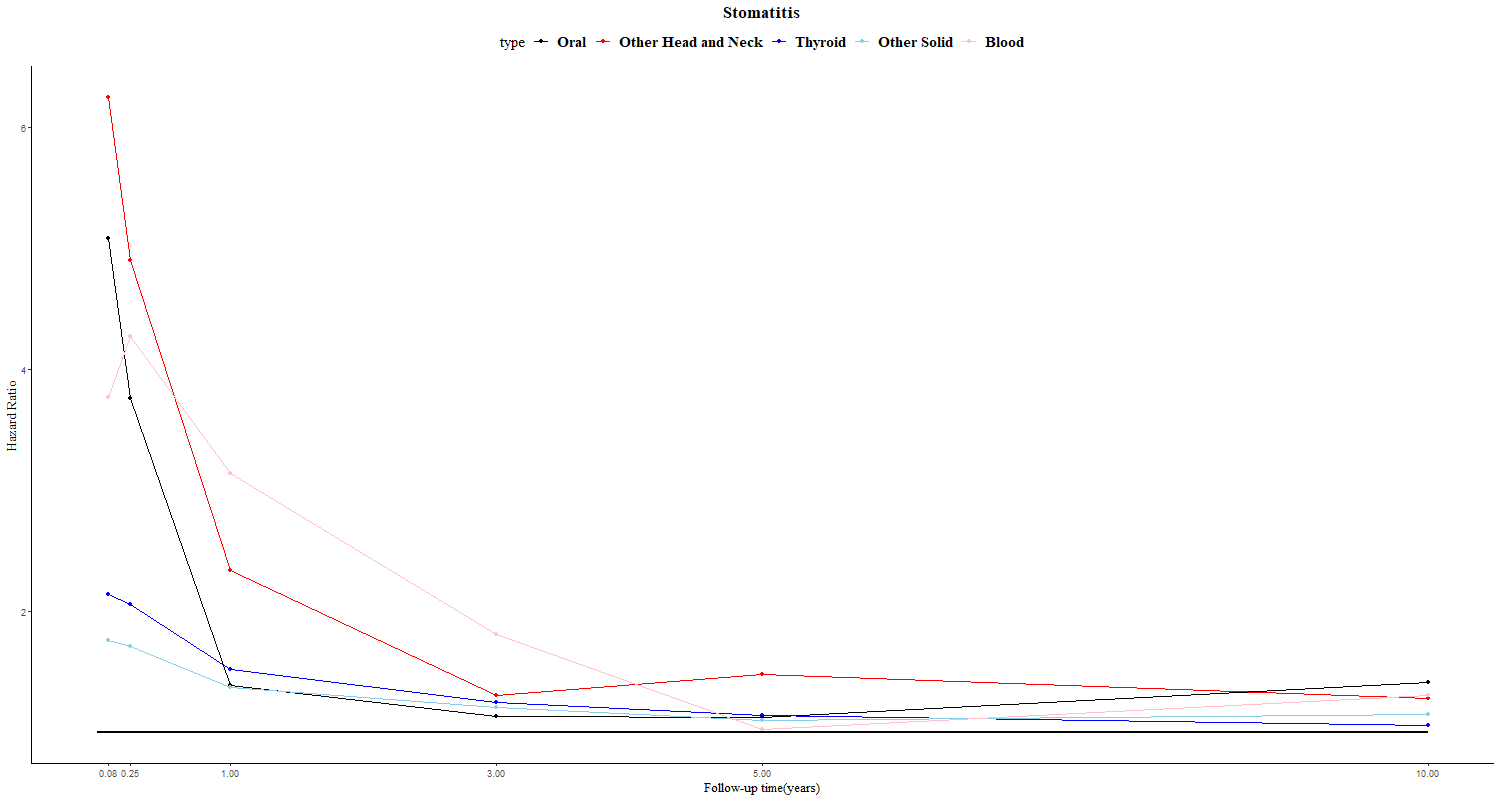


SHRs are expressed by points according to the follow-up period. Cancer types are illustrated by different-colored lines. The bold line represents a hazard ratio of 1.0, indicating the same risk between cancer patients and the control group.

Supplementary Figure 2. Time-dependent SHRs of treatment events for tooth loss considering death as a competing event depending on cancer type.


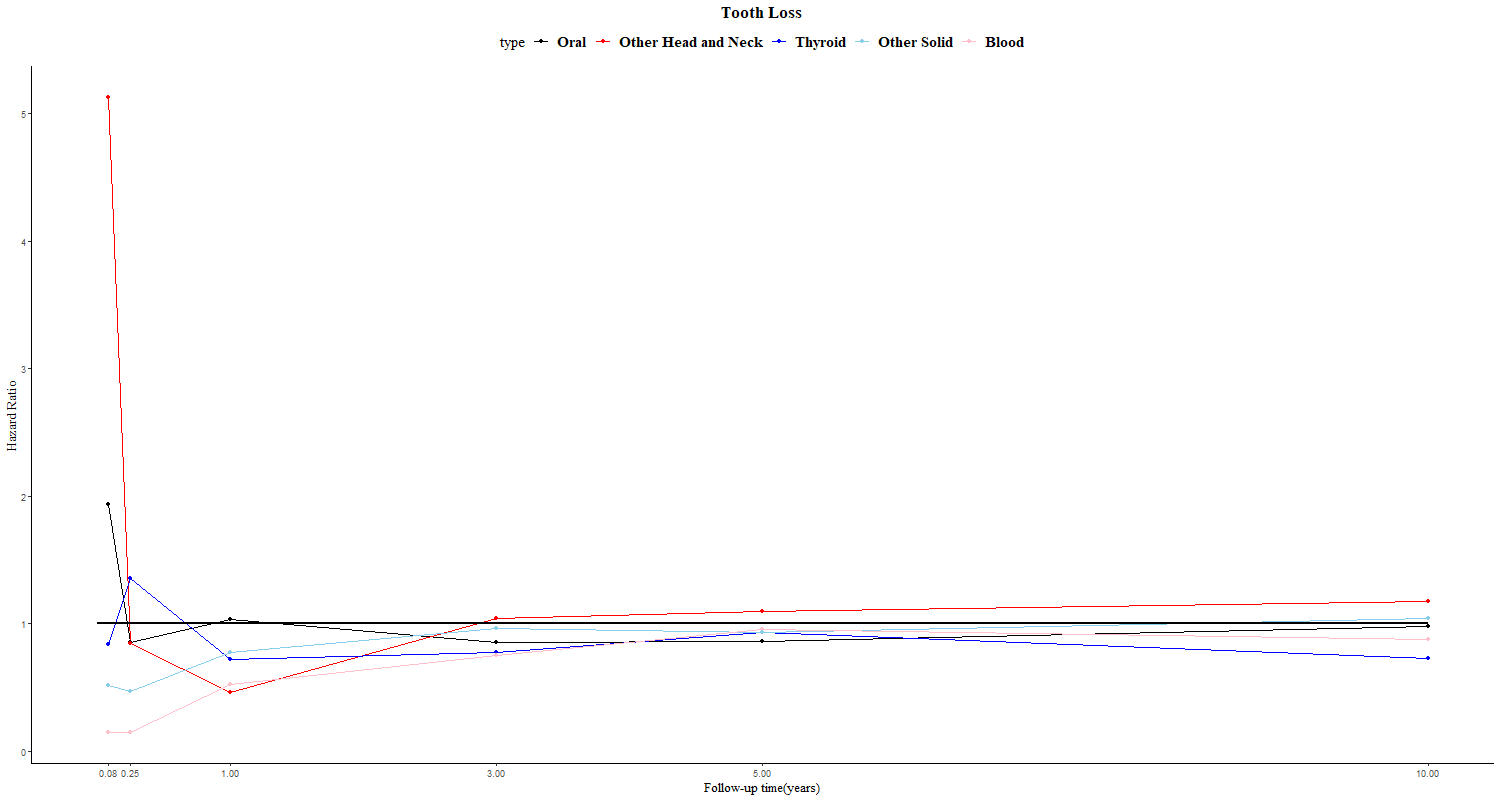


SHRs are expressed by points according to the follow-up period. Cancer types are illustrated by different-colored lines. The bold line represents a hazard ratio of 1.0, indicating the same risk between cancer patients and the control group.

**Supplementary 5. Time-dependent hazard ratios of treatment events for tooth loss considering death as a competing event depending on cancer type as expressed by points according to the follow-up period.** Cancer types are illustrated by different-colored lines. The bold line represents a hazard ratio of 1.0, indicating the same risk between cancer patients and the control group.

Supplementary Figure 3. Time-dependent SHRs of treatment events for dental caries/pulp diseases considering death as a competing event depending on cancer type.


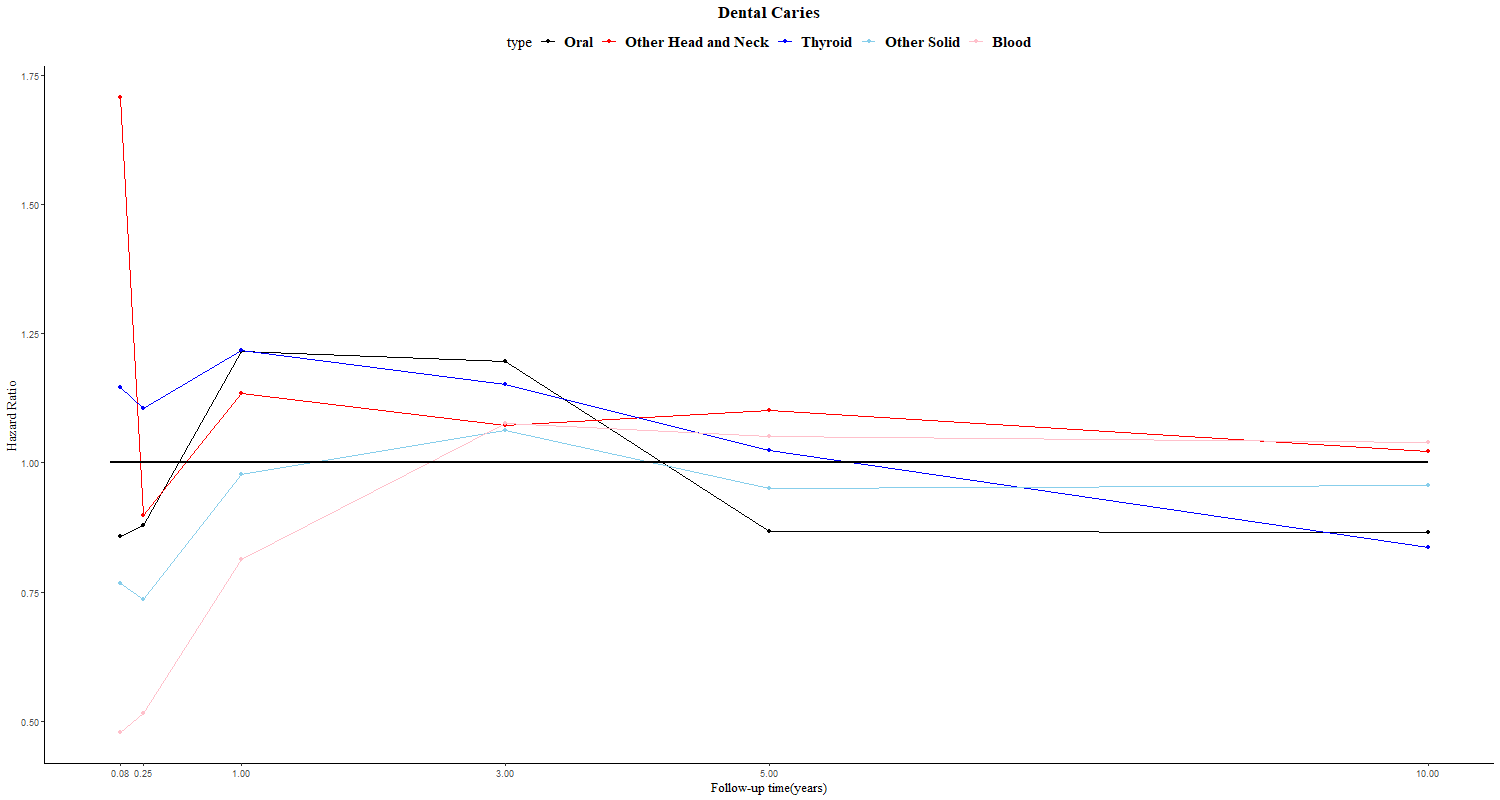


SHRs are expressed by points according to the follow-up period. Cancer types are illustrated by different-colored lines. The bold line represents a hazard ratio of 1.0, indicating the same risk between cancer patients and the control group.

Supplementary Figure 4. Time-dependent SHRs of treatment events for gingivitis/periodontal diseases considering death as a competing event depending on cancer type.


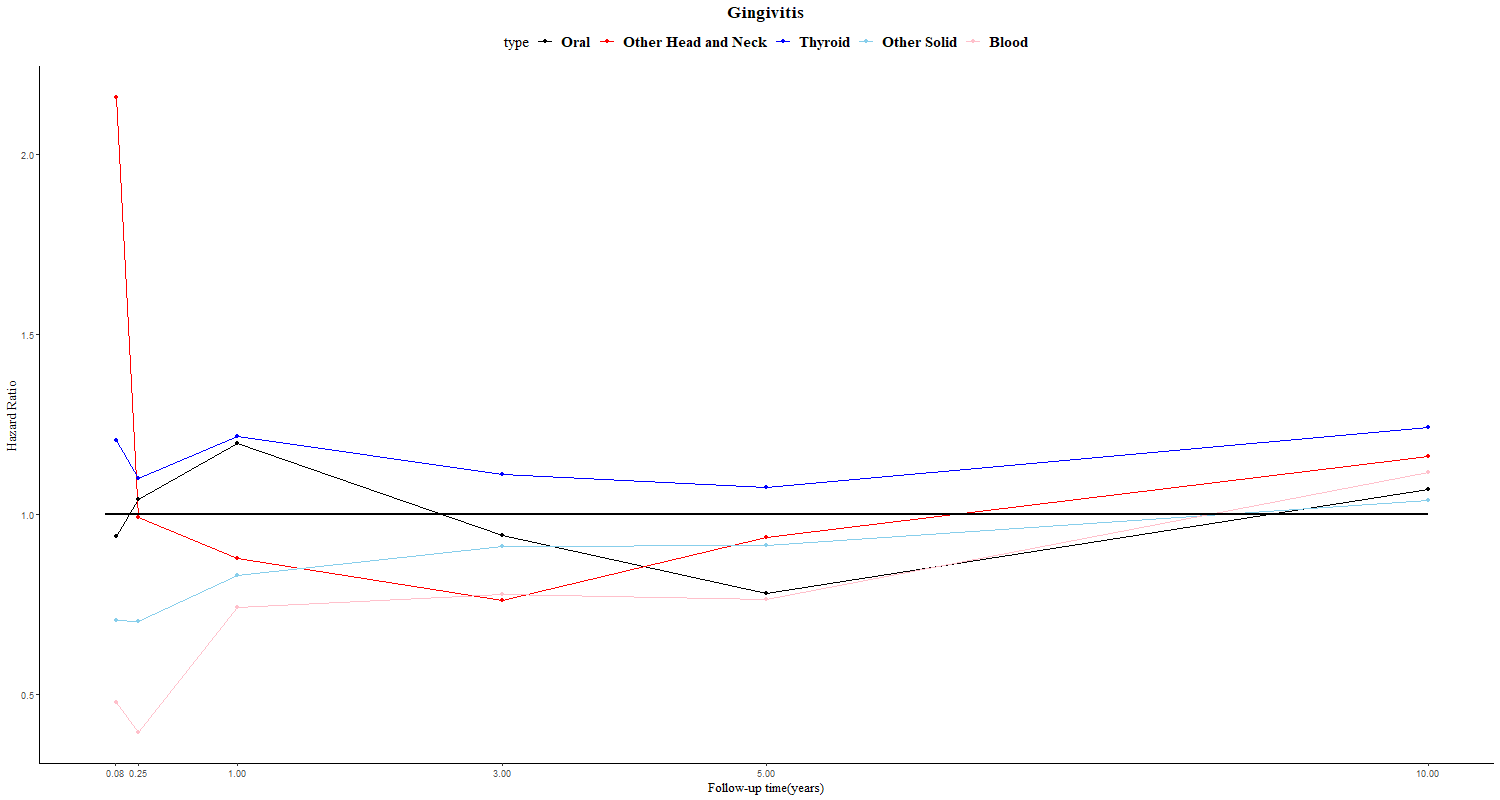


SHRs are expressed by points according to the follow-up period. Cancer types are illustrated by different-colored lines. The bold line represents a hazard ratio of 1.0, indicating the same risk between cancer patients and the control group.

**Supplementary 7. Time-dependent hazard ratios of treatment events for gingivitis/periodontal diseases considering death as a competing event depending on cancer type as expressed by points according to the follow-up period.** Cancer types are illustrated by different-colored lines. The bold line represents a hazard ratio of 1.0, indicating the same risk between cancer patients and the control group.

Supplementary Table 4. Time-dependent SHRs of major dental disease treatment events considering death as a competing event by cancer type

| Cancer type | Time | SHR (95% CI) | | | | | | | |
| --- | --- | --- | --- | --- | --- | --- | --- | --- | --- |
|  |  | Stomatitis | | Tooth loss | | Dental caries/  pulp disease | | Gingivitis/  periodontal disease | |
| Oral | < 30 d  30 d to 3 mo  3 mo to 1 y  1 y-3 y  3 y-5 y  > 5 y | 5.09  3.76  1.38  1.13  1.12  1.41 | (2.96-8.74)  (2.47-5.73)  (0.99-1.93)  (0.86-1.50)  (0.82-1.55)  (1.04-1.92) | 1.94  0.86  1.04  0.85  0.86  0.98 | (0.65-5.75)  (0.34-2.14)  (0.67-1.62)  (0.60-1.21)  (0.58-1.27)  (0.69-1.40) | 0.86  0.88  1.22  1.20  0.87  0.87 | (0.41-1.78)  (0.52-1.48)  (0.95-1.56)  (0.98-1.47)  (0.66-1.14)  (0.65-1.15) | 0.94  1.04  1.20  0.94  0.78  1.07 | (0.50-1.75)  (0.68-1.60)  (0.96-1.49)  (0.78-1.14)  (0.61-1.00)  (0.85-1.35) |
| Other head and neck | < 30 d  30 d to 3 mo  3 mo to 1 y  1 y to 3 y  3 y to 5 y  > 5 y | 6.25  4.90  2.34  1.31  1.48  1.28 | (3.75-10.43)  (3.31-7.27)  (1.71-3.19)  (0.94-1.80)  (1.00-2.17)  (0.79-2.05) | 5.12  0.85  0.46  1.04  1.10  1.18 | (2.30-11.42)  (0.30-2.38)  (0.23-0.91)  (0.70-1.55)  (0.68-1.78)  (0.68-2.05) | 1.71  0.90  1.14  1.07  1.10  1.02 | (1.02-2.87)  (0.55-1.47)  (0.87-1.48)  (0.85-1.35)  (0.82-1.48)  (0.71-1.48) | 2.16  0.99  0.88  0.76  0.94  1.16 | (1.49-3.11)  (0.69-1.43)  (0.69-1.11)  (0.62-0.94)  (0.73-1.21)  (0.86-1.56) |
| Thyroid | < 30 d  30 d to 3 mo  3 mo to 1 y  1 y to 3 y  3 y to 5 y  > 5 y | 2.14  2.06  1.52  1.25  1.14  1.05 | (1.59-2.87)  (1.66-2.55)  (1.33-1.74)  (1.11-1.39)  (0.99-1.31)  (0.88-1.26) | 0.84  1.36  0.72  0.78  0.93  0.73 | (0.38-1.83)  (0.85-2.15)  (0.54-0.97)  (0.64-0.96)  (0.75-1.17)  (0.55-0.98) | 1.15  1.11  1.22  1.15  1.02  0.84 | (0.91-1.45)  (0.93-1.32)  (1.11-1.34)  (1.07-1.24)  (0.93-1.13)  (0.72-0.97) | 1.21  1.10  1.22  1.11  1.08  1.24 | (1.00-1.45)  (0.96-1.27)  (1.13-1.31)  (1.04-1.18)  (0.99-1.17)  (1.12-1.38) |
| Blood | < 30 d  30 d to 3 mo  3 mo to 1 y  1 y to 3 y  3 y to 5 y  > 5 y | 3.77  4.27  3.14  1.81  1.02  1.30 | (2.67-5.32)  (3.29-5.56)  (2.51-3.93)  (1.41-2.33)  (0.68-1.53)  (0.84-2.02) | 0.15  0.15  0.53  0.75  0.96  0.88 | (0.04-0.61)  (0.04-0.61)  (0.31-0.91)  (0.51-1.12)  (0.60-1.53)  (0.46-1.67) | 0.48  0.52  0.81  1.08  1.05  1.04 | (0.26-0.88)  (0.34-0.79)  (0.66-1.01)  (0.90-1.29)  (0.82-1.35)  (0.74-1.46) | 0.48  0.40  0.74  0.78  0.76  1.12 | (0.29-0.78)  (0.27-0.59)  (0.61-0.90)  (0.66-0.92)  (0.60-0.98)  (0.87-1.44) |
| Other solid | < 30 d  30 d to 3 mo  3 mo to 1 y  1 y to 3 y  3 y to 5 y  > 5 y | 1.76  1.71  1.37  1.21  1.09  1.15 | (1.18-2.62)  (1.19-2.45)  (1.24-1.51)  (1.14-1.28)  (1.01-1.18)  (1.05-1.26) | 0.52  0.47  0.78  0.96  0.93  1.04 | (0.32-0.59)  (0.32-0.49)  (0.63-0.77)  (0.88-1.01)  (0.86-1.05)  (1.00-1.23) | 0.77  0.74  0.98  1.06  0.95  0.96 | (0.68-0.87)  (0.66-0.82)  (0.93-1.03)  (1.02-1.11)  (0.90-1.01)  (0.89-1.03) | 0.70  0.70  0.83  0.91  0.91  1.04 | (0.64-0.78)  (0.65-0.75)  (0.80-0.86)  (0.88-0.94)  (0.87-0.96)  (0.98-1.10) |
